# A Conserved Function in Phosphatidylinositol Metabolism for Mammalian Vps13 Family Proteins

**DOI:** 10.1371/journal.pone.0124836

**Published:** 2015-04-27

**Authors:** Jae-Sook Park, Simon Halegoua, Shosei Kishida, Aaron M. Neiman

**Affiliations:** 1 Department of Biochemistry and Cell Biology, Stony Brook University, Stony Brook, New York, 11794–5215, United States of America; 2 Department of Neurobiology and Behavior, Stony Brook University, Stony Brook, New York, 11794–5230, United States of America; 3 Graduate School of Medical and Dental Sciences, Kagoshima University, Kagoshima, 890–8544, Japan; Institut Jacque Monod, Centre National de la Recherche Scientifique, FRANCE

## Abstract

The Vps13 protein family is highly conserved in eukaryotic cells. In humans, mutations in the gene encoding the family member *VPS13A* lead to the neurodegenerative disorder chorea-acanthocytosis. In the yeast *Saccharomyces cerevisiae*, there is just a single version of *VPS13*, thereby simplifying the task of unraveling its molecular function(s). While *VPS13* was originally identified in yeast by its role in vacuolar sorting, recent studies have revealed a completely different function for *VPS13* in sporulation, where *VPS13* regulates phosphatidylinositol-4-phosphate (PtdIns(4)P) levels in the prospore membrane. This discovery raises the possibility that the disease phenotype associated with *vps13A* mutants in humans is due to misregulation of PtdIns(4)P in membranes. To determine whether *VPS13A* affects PtdIns(4)P in membranes from mammalian neuronal cells, phosphatidylinositol phosphate pools were compared in PC12 tissue culture cells in the absence or presence of *VPS13A*. Consistent with the yeast results, the localization of PtdIns(4)P is specifically altered in *VPS13A* knockdown cells while other phosphatidylinositol phosphates appear unaffected. In addition, *VPS13A* is necessary to prevent the premature degeneration of neurites that develop in response to Nerve Growth Factor. The regulation of PtdIns(4)P is therefore a conserved function of the Vps13 family and may play a role in the maintenance of neuronal processes in mammals.

## Introduction

*Saccharomyces cerevisiae VPS13* is the founding member of a protein family that is highly conserved in eukaryotic cells[1]. Despite its extensive conservation, little is known about the molecular function of this protein family. In yeast, *VPS13* was originally identified by the failure of *vps13* mutant cells to deliver a lumenal protease to the vacuole (the yeast lysosome) [2]. This defect appears to be a secondary consequence of the failure of *vps13* mutants to properly recycle sorting receptors from the endosome to the Golgi complex [3]. In addition to this role in vesicle transport, *VPS13* has a second function in the process of sporulation [4–6].

Sporulation is the yeast equivalent of gametogenesis [7]. During this process, the four haploid nuclei produced by meiosis are each enveloped within a novel intracellular membrane called the prospore membrane. Closure of each prospore membrane around a nucleus delimits the spore and the prospore membrane then serves as the plasma membrane of the spore. In sporulating cells, Vps13 binds to the sporulation-specific protein Spo71 and translocates from the endosome to the prospore membrane [6]. Loss of *VPS13* leads to morphological defects of the membrane [4,5]. Examination of fluorescent reporters for different lipid head groups shows that prospore membranes in *vps13* cells have reduced levels of phosphatidylinositol-4-phosphate (PtdIns(4)P), phosphatidylinositol-4,5-bisphosphate (PtdIns(4,5)P_2_) and phosphatidic acid. Reduction of the latter two lipids can be accounted for by an effect on PtdIns(4)P levels, and reduction in these different lipids is likely the cause of the abnormal membrane morphologies [5].

In humans, there are four genes encoding Vps13 family members; *VPS13A*, *VPS13B*, *VPS13C*, and *VPS13D*[1]. *VPS13A* is of particular interest, as loss of this gene results in the neurodegenerative disorder, chorea-achanthocytosis (ChAc). ChAc is characterized by involuntary movements, including chorea and dystonia, as well as the presence of misshapen red blood cells (acanthocytes)[8,9]. The product of the *VPS13A* gene is called chorein and disease alleles of *VPS13A* lead to loss of the chorein protein [10,11].

Despite its conservation, little is known about the function of chorein. The protein has been localized to the Golgi in some cell types and an effect on actin organization in *vps13A* mutant cells has been reported [12–16], however the function(s) of the mammalian family members remains obscure. Furthermore, although the loss of chorein causes neuronal degeneration in humans and in mouse models, how loss of *VPS13A* leads to neurodegeneration is unknown [17,18]. The PC12 cell line has been a useful model with which to study the elaboration of key neuronal phenotypes, mediated by nerve growth factor (NGF) treatment [19]. For example, NGF induces the *de novo* outgrowth of neuritic processes, analogous to axon and dendrite growth[19]. To gain insights into both the role of chorein in mammalian cells and its critical role in neuronal function, we have used shRNA mediated knockdown of *VPS13A* in the neuronal model PC12 cell line to test whether *VPS13A* regulates PtdIns phosphate pools analogous to the yeast ortholog. *VPS13A* knockdown results in a reduction of of PtdIns(4)P in the Golgi and plasma membrane without obvious changes to the plasma membrane levels of PtdIns(4,5)P_2_ or PtdIns(3,4,5)P_3_. In addition, *VPS13A* is required to prevent the premature degeneration of elongating neurites. These results suggest that regulation of PtdIns(4)P is a conserved function of Vps13 family proteins and provide potential insight into the basis for neuronal degeneration seen in ChAc.

## Materials and Methods

### Plasmids

Plasmids used in this study are listed in Table 1. The pRFP-C-RS vectors used for transient expression of different shRNAs were purchased from OriGene (TF704233, RefSeq NM_001100975) (Rockville, MD, USA). These vectors express both the red fluorescent protein (RFP) and short hairpin RNAs (shRNAs) that can mediate gene knockdown *via* the RNAi pathway. To stably express these shRNAs from recombinant lentivirus the shRNA genes were moved from the pRFP-C-RS plasmids into pHAGE vectors [20] to allow for packaging into lentiviral particles. These plasmids were made in several steps. First, the *GFP* gene was deleted from the vector pHAGE-Ubc-GIR [20]by digestion with NotI and NdeI followed by filling of the blunt ends using the Klenow fragment of DNA polymerase I (New England Biolabs, Ipswich, MA, USA) and religation to generate pJS66. The different shRNA genes with their promoters, or the multiple cloning site alone, were then amplified using the polymerase chain reaction (PCR) from the different pRFP-C-RS plasmids using primers, JSO197 (GgactagtGAATTCCCCAGTGGAAAGAC) and JSO198 (GgactagtAAGCTTTTCCAAAAAAGTCTTCT). The PCR products were digested with SpeI and ligated into similarly digested pJS66, creating JS67, JS68, JS69 and JS70, carrying the RFP gene as well as a polylinker, the scrambled shRNA, shRNA3 or shRNA5, respectively, in the pHAGE backbone.

**Table 1:** Plasmids.

Name	Relevant Genes Expressed	Source
pRFP-C-RS-vector 14	RFP	OriGene TR30014
pRFP-C-RS-scrambled shRNA 15	RFP, scrambled shRNA	OriGene TR30015
pRFP-C-RS-*VPS13A* shRNA 3	RFP, *VPS13A* shRNA 3	OriGene FI716933
pRFP-C-RS-*VPS13A* shRNA 4	RFP, *VPS13A* shRNA 4	OriGene FI716934
pRFP-C-RS-*VPS13A* shRNA 5	RFP, *VPS13A* shRNA 5	OriGene FI716935
pRFP-C-RS-*VPS13A* shRNA 6	RFP, *VPS13A* shRNA 6	OriGene FI716936
pHAGE-Ubc-GIR	RFP, GFP	[20]
pJS66	RFP	This study
pJS67	RFP	This study
pJS68	RFP, scrambled shRNA	This study
pJS69	RFP, *VPS13A* shRNA 3	This study
pJS70	RFP, *VPS13A* shRNA 5	This study
pEGFP-C1-OSBP-PH	GFP-OSBP-PH	[24]
pEGFP-N1-PLCδ1-PH	PLCδ1-PH-GFP	[25]
pEGFP-N1-AKT-PH	Akt-PH-GFP	[25]

### PC12 cell culture, transfection and neurite growth

PC12 cells were grown on tissue culture dishes in Dulbecco’s modified eagle medium (DMEM) supplemented with 5% fetal bovine serum (FBS) and 10% horse serum (HS)[21]. For transfection, PC12 cells were seeded in growth medium at about 4x10^4^ cells in 60-mm culture dishes (day 1) and incubated at 37°C with 10% CO_2_. On day 3, the medium was replaced with Opti-MEM Reduced Serum Medium (Gibco, Grand Island, NY, USA) containing 10% HS and 5% FBS. When PC12 cells reached 80% confluency (day 4), plasmids were transfected into the cells using Lipofectamine 2000 (Invitrogen, Carlsbad, CA, USA) according to the company protocol. On day 5, cells were transferred onto cover slips pre-treated with poly-L-Lysine (Sigma-Aldrich, St. Louis, MO, USA) for further manipulation and/or microscopic visualization. Neurite growth was assayed as described [19]. Briefly, cells were seeded at low density (about 2x10^3^ cells/cm^2^) in growth medium. NGF (final concentration 50ng/ml) was added on day 1 and day 3 after seeding. Plates were fixed for microscopy on days 0, 3, and 5. Neurite morphology was scored when the extensions were at least twice the length of the cell body. Blebs are defined as large spherical protuberances along the neurite.

### HEK 293-T cell culture and lentivirus production

A standard method for recombinant lentiviral particle production was used[20,22]. Briefly, on day 1, HEK 293-T cells[23], were seeded in DMEM containing 10% FBS and 10 units/ml penicillin and streptomycin (Gibco, Grand Island, NY, USA) at density 3x10^6^ cells per 10-cm culture dish. The next day, the medium was changed to DMEM containing 10% FBS without antibiotics at least 1 hour prior to transfection. To produce lentivirus carrying various shRNAs, HEK 293-T cells were co-transfected with the pJS plasmids carrying the shRNAs and plasmids carrying individual lentiviral packaging components using LipoD293 (SignaGen, Rockville, MD, USA)[22]. To collect the lentiviral particles, on days 3 and 4, the medium was removed from each plate, stored at 4°C for later concentration, and replaced with fresh medium. The media from days 3, 4 and 5 were pooled and passed through a 0.4 μm syringe-tip filter to get rid of debris and the viral particles were then concentrated by centrifugation at 100,000 x g for 2 hours using a SW28 rotor in a Beckman Optima XL-100K Ultracentrifuge. The concentrated lentiviral particles were resuspended in 1 mL of DMEM without serum and stored at -80°C.

To produce PC12 cells stably expressing shRNAs, 400 μl of the concentrated lentiviral particles were mixed with 6 μg/mL polybrene (Sigma-Aldrich, St. Louis, MO, USA) and directly added onto the plated PC12 cells. The infected cells were then incubated at 37°C in 10% CO_2_ for 2 hours with agitation every 20 minutes. After incubation, the virus particle mixture was replaced with DMEM supplemented with 5% FBS and 10% HS.

### Western blot analysis

Recombinant lentivirus infected PC12 cells were grown up to 80% confluent density in 100-mm culture dishes and collected for total protein extraction by centrifugation at 100 x g for 5 minutes. The PC12 cells were then resuspended in 100 μL lysis buffer (10mM Tris-HCl pH7.5; 150 mM NaCl; 0.5 mM EDTA; 0.5% NP-40) containing 1mM PMSF and a protease inhibitor cocktail (complete tablets, Mini EDTA-free, EASYpack) (Roche, Indianapolis, IN, USA). After incubation on ice for 30 minutes, cells were resuspended/lysed by vortexing and debris removed by centrifugation at 11000 x g for 5 minutes. The protein concentration of the lysate was measured by using the bicinchoninic acid (BCA) protein assay (Thermo Scientific, Rockford, IL, USA). Lysates were diluted with 5 X SDS sample buffer (25 mM Tris-HCl, 10% sodium-dodecyl-sulfate (SDS), 50% glycerol, 0.5mg/ml Bromophenol blue, 1.8 M 2-mercaptoethanol) to a final protein concentration of 8 μg/μL and boiled for 5 minutes.

Proteins were detected by western blot analysis using anti-Chorein [14]or anti-GAPDH (Calbiochem, Darmstadt, Germany) antibodies at 1:6000 or 1:2000 dilutions, respectively. Due to the large size of Chorein (350kDa), 3 to 8% NuPAGE Tris-Acetate gradient gels (Life Technologies, Carlsbad, CA, USA) were used. To transfer this large protein onto polyvinylidene fluoride (PVDF) membranes, wet transfer was conducted at 50 mA, overnight at 4°C.

### Microscopy

To examine the localization of the various GFP tagged pleckstrin homology (PH) domains, PC12 cells were grown on poly-L-Lysine coated cover slides and fixed with 3.7% paraformaldehyde (Electron Microscopy Sciences, Fort Washington, PA, USA). The fixed cells were imaged using a Zeiss Observer.Z1 microscope with an attached Orca II ERG camera (Hamamatsu, Bridgewater, NJ, USA). Zeiss Axiovision 4.8 software and ZEN 2012 blue edition software were used to acquire images. GFP localization was counted from cells exhibiting a RFP signal, indicating the presence of the shRNA vector. For quantitation of GFP fluorescence, images were analyzed with Image J software (NIH). For measurement of plasma membrane fluorescence, a line was drawn along the arc of the plasma membrane and the average pixel intensity along the line was used as the fluorescence value. For measurement of Golgi fluorescence, an outline was drawn around the Golgi elements and the mean intensity for all pixels within that area was used. Background fluorescence was subtracted from all images prior to measurement using the automated function within Image J.

### Immunostaining

To simultaneously visualize OSBP-GFP-PH with Golgi or nuclear markers, PC12 cells were grown on poly-L-Lysine coated cover slides and fixed with 3.7% paraformaldehyde (Electron Microscopy Sciences, Fort Washington, PA, USA). After washing with 1xPBS, these cells were permeabilized with 0.5% Triton X-100 in 1x PBS for 5 minutes and then washed three times for 5 minutes each, with 1x PBS. Blocking was performed with 10% goat serum in 1x PBS for 30 minutes. These permeablized cells were incubated with anti-GM130 (BD Biosciences, San Jose, USA) antibodies at 1: 100 dilution for 1 hour. After three washes with 1x PBS, Alexa-546-labeled anti-mouse IgG at a 1: 300 dilution was added to the cells for 1 hour. After one wash with 1x PBS, cells were incubated in 1ug/mL 4,6-diamidino-2-phenylindole (DAPI) in 1x PBS for 5 minute, washed three times with 1x PBS and sealed under a cover slip.

## Results

### Biosensors for PtdIns(4)P, PtdIns(4,5)P_2_ or PtdIns(3,4,5)P_3_ are found at the plasma membrane in PC12 cells

**Fig 1:**
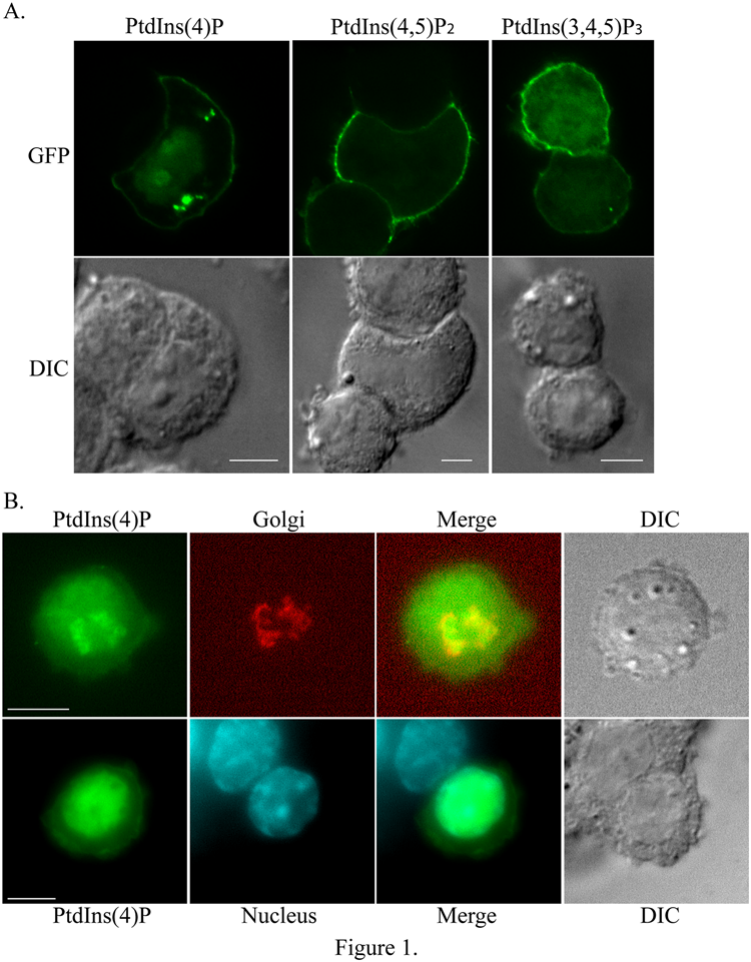
Localization of PtdIns(4)P, PtdIns(4,5)P2 or PtdIns(3,4,5)P3 in PC12 cells. (A) PC12 cells were transiently transfected with GFP-OSBP-PH, PLCδ1-PH-GFP or Akt-PH-GFP to visualize PtdIns(4)P, PtdIns(4,5)P_2_ or PtdIns(3,4,5)P_3_, respectively. (B) PC12 cells were transfected with GFP-OSBP-PH to visualize PtdIns(4)P, fixed and stained with antibodies to the *cis*-Golgi marker GM130 and with DAPI. Representative images are shown. At least 15 cells were scored for each transfection. Scale bars = 5μm.

A previous study demonstrated that *VPS13A* is expressed in neuron-like rat PC12 cells[14]. This cell line was therefore used to determine the role of *VPS13A* on the distribution of PtdIns phosphate pools. Although it is difficult to localize various phospholipids directly within a cell, this can be accomplished indirectly by determining the localization of proteins that interact specifically with these lipids and therefore act as biosensors that can be followed using microscopy[24,25]. PC12 cells were first transfected with plasmids expressing GFP reporters that act as biosensors for either PtdIns(4)P (GFP-OSBP-PH), PtdIns(4,5)P_2_ (PLCδ1-PH-GFP), or PtdIns(3,4,5)P_3_ (Akt-PH-GFP)[24,25]. Consistent with previous reports describing the localization of these same reporters in other cell types, PtsIns(4,5)P_2_ and PtdIns(3,4,5)P_3_ reporters localized primarily to the plasma membrane, whereas, in addition to plasma membrane localization the PtdIns(4)P reporter displayed bright perinuclear signals and variable nuclear fluorescence as well as plasma membrane localization [24–26] (Fig 1A). The nuclear and Golgi localization was confirmed by costaining of cells with DAPI or the cis-Golgi marker GM130, respectively (Fig 1B). Thus, PC12 cells have significant pools of PtdIns(4)P in both the Golgi and the plasma membrane.

### PtdIns(4)P is reduced in *VPS13A*-knockdown PC12 cells

**Fig 2:**
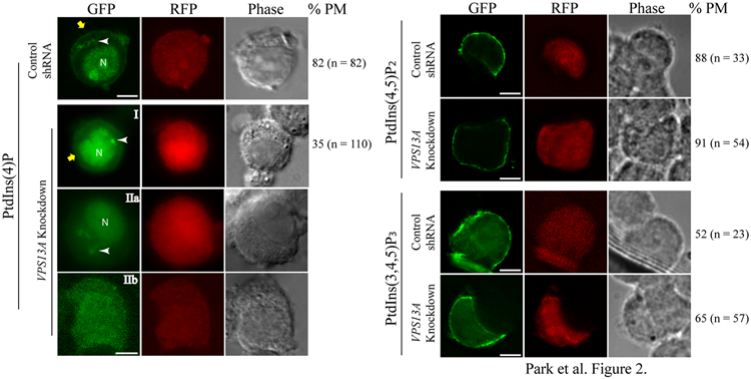
Localization of PtdIns(4)P, PtdIns(4,5)P2 or PtdIns(3,4,5)P3 in PC12 cells carrying scrambled shRNA or *VPS13A* knockdown shRNA. PC12 cells were transiently co-transfected with GFP tagged PH domains and vectors expressing either a control, scrambled shRNA or *VPS13A* shRNA3. Red fluorescence indicates the presence of the shRNA vector. For each sensor, the percentage of knockdown cells in which the sensor was localized at the plasma membrane and the number (n) of cells scored are shown. The data are pooled from three experiments for the PtdIns(4)P and PtdIns(4,5)P_2_ sensor and two experiments for the PtdIns(3,4,5)P_3_ sensor. In *VPS13A* knockdown cells, three patterns of localization for the PtdIns(4)P sensor are seen: class I are similar to the control cells (indicated by “I”); class IIa are lacking in plasma membrane fluorescence but have clear Golgi and nuclear fluorescence (IIa); and class IIb show diffuse fluorescence throughout the cell (IIb). Yellow arrows indicate the plasma membrane. White arrowheads indicate Golgi elements. N = nucleus. Scale bars = 5μm.

**Fig 3:**
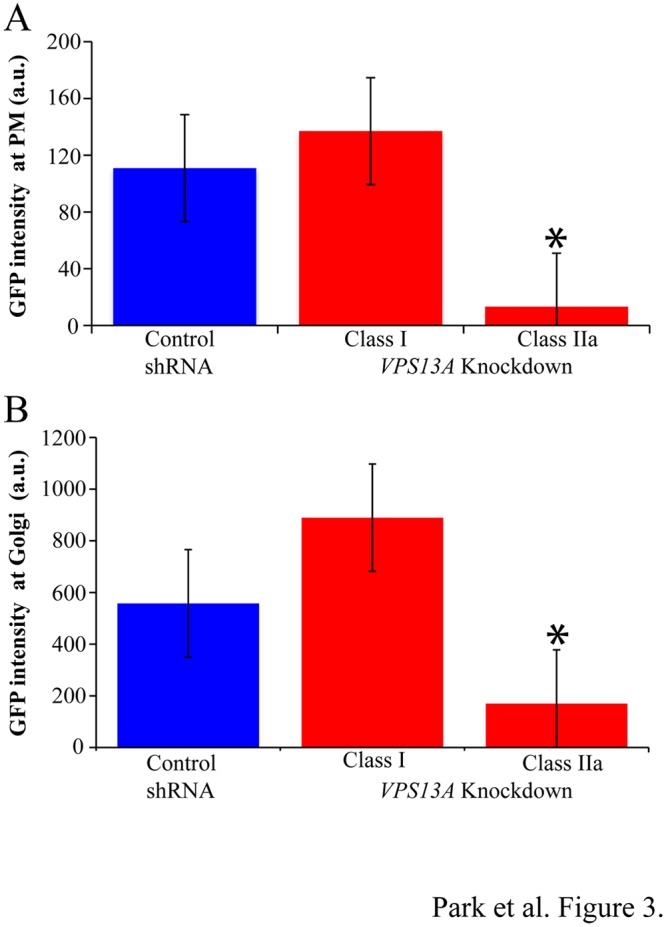
Quantitation of PtdIns(4)P within plasma membranes and the Golgi complex in *VPS13A* knockdown cells. (A) The average fluorescence intensity of the GFP-OSBP-PH from the plasma membrane was quantified from cells cotransfected with the control, scrambled shRNA, or the *VPS13A* knockdown shRNA3. Fluorescence was measured separately for knockdown cells in class I and class IIa (described in legend to Fig 2). Class IIa cells show a significant decrease in plasma membrane fluorescence relative to control (p<0.001, student’s t-test). Error bars indicate standard error. Greater than 12 cells scored in all classes. a.u. = arbitrary units. (B) Average fluorescence intensity of GFP-OSPB-PH from the Golgi complex quantified as in (A). Asterisk indicates significantly reduced fluorescence in the Class IIa cells relative to the control (p<0.01, student’s t-test).

To see whether *VPS13A* affects the localization of PtdIns(4)P in mammalian cells, PC12 cells were transiently co-transfected with the lipid reporter constructs and a plasmid expressing an shRNA to knockdown *VPS13A* expression. Four different commercially available shRNAs targeting rat *VPS13A* were tested (see Materials and Methods). As a control, co-transfections were also performed with a plasmid in which the *VPS13A* shRNA target sequence was scrambled and therefore should not affect chorein protein levels. The shRNA vectors also express RFP, so co-transfected cells can be identified by the presence of both GFP and RFP fluorescence. Only cells displaying both RFP and GFP signals were examined. No differences in the distribution of the PtdIns(4,5)P_2_ and PtdIns(3,4,5)P_3_ sensors were seen when cells expressing the *VPS13A* knockdown shRNA3 were compared with cells expressing the scrambled shRNA or with cells transfected only with the GFP reporter (Fig 2). By contrast, the localization of GFP-OSBP-PH was altered in *VPS13A* knockdown cells relative to the control cells. Two different classes of cells were seen after knockdown; cells that appeared wild type in distribution of the GFP-OSBP-PH (class I) and cells that lacked clear plasma membrane signal from the reporter (class II). Within this second class, about half the cells still showed detectable Golgi and nuclear fluorescence (class IIa), while the remainder displayed GFP fluorescence diffusely throughout the cell (class IIb) (Fig 2). To examine these results more quantitatively, the fluorescence intensity of the GFP signals was measured. When the GFP intensity at the plasma membrane or at the Golgi in cells transfected with the control shRNA was compared to the class 1 knockdown cells, no significant differences in fluorescence intensity was seen. By contrast, the signal intensity from the plasma membrane was reduced to background levels and fluorescence from the Golgi was significantly lower in class IIa cells (Fig 3). Because specific organelles could not be identified in class IIb cells, they were omitted from this analysis. A similar effect was seen with two additional shRNA knockdown constructs (J.S.P., unpublished obs.). These results indicate that knockdown of *VPS13A* leads to reduction of PtdIns(4)P levels in both the Golgi and the plasma membrane in a majority of knockdown cells.

**Fig 4:**
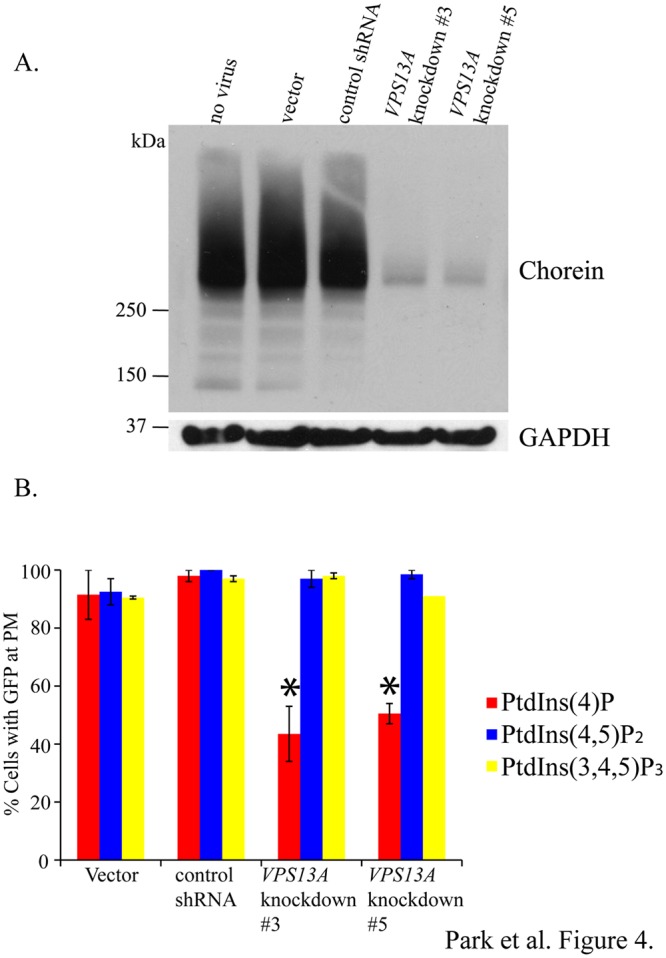
Efficacy of the *VPS13A* knockdown shRNAs in lentivirus infected cells. (A) Western blot using anti-chorein antibodies to determine the level chorein expression in control and lentivirus-infected knockdown cells. The levels of GAPDH were used as a loading control for each lysate. For chorein and GAPDH detection, 100μg and 50μg of total lysates were loaded, respectively. (B) Quantitation of PtdIns-phosphate sensor localization to the plasma membrane in in control and lentivirus-infected knockdown cells. Two independent experiments were performed for each reporter. At least 50 cells scored in each experiment. Error bars indicate one standard deviation. Asterisks indicate a significant reduction of PtdIns(4)P sensor localization to the plasma membrane in cells expressing knockdown shRNA #3 or #5 relative to those expressing the scrambled shRNA (p<0.0001, Chi square test).

The co-transfection efficiency of the PC12 cells was low (<10%), making it difficult to assess the efficacy of the knockdown vectors in reducing *VPS13A* expression. To circumvent these problems two *VPS13A* knockdown shRNAs (shRNA3 and shRNA5) and the scrambled control shRNA were subcloned into lentiviral vectors and these vectors were used to generate stable PC12 cell lines expressing the shRNAs. Western blot analysis using antibodies against the chorein protein [14] demonstrated that the knockdown shRNAs greatly reduced the levels of chorein (Fig 4A), confirming the efficacy of the knockdown constructs.

The knockdown and scrambled shRNA cell lines were then transfected with the three different lipid biosensor constructs and the localization of the GFP reporters was examined. As in the transient transfection experiments, no major differences in distribution of the PtdIns(4,5)P_2_ or PtdIns(3,4,5)P_3_ reporters were observed in the *VPS13A* knockdown cell lines compared to the controls. In contrast, the fraction of cells displaying plasma membrane fluorescence of the PtdIns(4)P reporter was significantly reduced (p<0.0001, Chi square test) in the knockdown cell lines (Fig 4B). Thus, the same partial effect on PtdIns(4)P distribution is seen in stable and transient transfections of the knockdown shRNA, indicating that this partial penetrance is likely true of a complete knockdown. This is reminiscent of results in yeast, where deletion of *VPS13* causes loss of recruitment of the PtdIns(4)P reporter to about 70% of prospore membranes [5].

### *VPS13A* knockdown causes abnormal neurite growth

**Fig 5:**
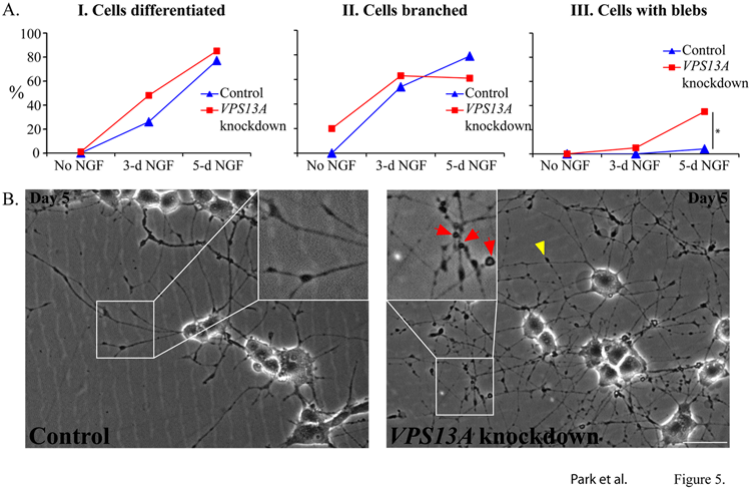
Differentiation and degeneration of *VPS13* knockdown PC12 cells upon treatment with NGF. (A) NGF treated-PC12 cells infected with scrambled or *VPS13A* shRNAs were fixed on the indicated day and scored for the presence of extended neurites (indicating differentiation, left graph), the fraction of neurites that were branched (middle graph), or the presence of blebs on the neurites. Asterisk indicates a significant difference in the frequency of blebs (p<0.001, Chi square test). The time course was performed twice with similar results, data from one experiment is shown. More than 60 cells were scored for each feature at each time point. (B) Neurite images from lentivirus infected PC12 cells 5 days after NGF treatment. Small boxes are magnified to show the neurites. Red arrows indicate blebs and yellow arrowhead indicates the degenerating neurite in PC12 cells carrying *VPS13A* shRNA 3Scale bar = 50μm.

PC12 cells elaborate a sympathetic neuron phenotype when NGF is added to the culture medium[27,28]. Over the course of several days, the cells extend multiple neurites with characteristic growth cones at their tips[21]. The stably transfected PC12 cells were used to test the possible impact of *VPS13A* knockdown on NGF-directed neurite growth. Both lines carrying scrambled or *VPS13A* shRNAs were sparsely seeded on poly-lysine coated glass cover slips and NGF was added to the medium. At intervals, cells were imaged by phase contrast microscopy and scored for neurite number, length, branching and morphology. Knockdown of *VPS13A* caused no evident change in the fraction of cells that elaborated neurites or in the number, length or growth cone morphology of the neurites produced, suggesting that *VPS13A* is not required for NGF-mediated neurite outgrowth (Fig 5). The *VPS13A* knockdown cells did, however, display morphological abnormalities of the neurites after several days of growth in NGF that were not observed in the controls. Specifically, large spherical protuberances or 'blebs' could be seen along the length of the neurites. These blebs were abundant by 5 days after induction and were coincident with neurite degeneration. These observations suggest that *VPS13A* is important for the maintenance of normal neurite morphology and function.

## Discussion

This study describes evidence that the function of yeast *VPS13* in regulation of PtdIns(4)P levels is conserved in the mammalian *VPS13A*. As in sporulating yeast cells, loss of *VPS13A* function causes reduction in intracellular PtdIns(4)P. In sporulating yeast, the *VPS13*-sensitive PtdIns(4)P pool is in the prospore membrane, which is a developing plasma membrane. In the neuronal PC12 cell model, PtdIns(4)P levels in both the Golgi and the plasma membrane are sensitive to *VPS13A* knockdown, though the effect is stronger at the plasma membrane. Thus, not only the activity but the pool of PtdIns(4)P regulated is conserved. Notably, in both mammalian and yeast cells, the effect on the levels of PtdIns(4)P is somewhat modest. The fraction of cells that lose localization of the GFP reporter for PtdIns(4)P is reduced two- to five-fold (Fig 3;[5]). This indicates that PtdIns(4)P is not eliminated, but the concentration in these respective membranes is reduced to a range that effects recruitment of the GFP reporter.

The drop in PtdIns(4)P levels in the prospore membrane of a *vps13Δ* mutant causes a corresponding decrease in the product lipid PtdIns(4,5)P_2_[5]. In contrast, no reduction of either PtdIns(4,5)P_2_ or PtdIns(3,4,5)P_3_ were observed in *VPS13A* knockdown cells. Using the same lipid biosensors used here, mouse embryonic fibroblasts lacking the PtdIns-4-kinase PI4KIIIα were shown to lose both PtdIns(4)P and PtdIns(4,5)P_2_ at the plasma membrane [29]. Interestingly, however, total PtdIns(4,5)P_2_ was not as strongly reduced in the PI4KIIIα knockout cells because of compensatory synthesis through upregulation of PtdIns(4)P 5-kinases[29]. It may be that the effect of *VPS13A* knockdown on PtdIns(4)P is modest enough that these compensatory pathways can mitigate effects on PtdIns(4,5)P_2_ levels in PC12 cells.

Studies of *VPS13A* knockdown in K562, HEK 293 and vascular endothelial (HUVEC) cell lines as well as erythrocytes from ChAc patients have suggested a role for *VPS13A* in regulation of the actin cytoskeleton[12–16]. In these conditions, the ratio of soluble actin over filamentous actin increased and the filamentous actin network was reduced, indicating that *VPS13A* reduction causes actin depolymerization. Moreover, chorein physically interacts and partially colocalizes with β-actin in HEK 293 cells[16]. While regulation of actin may be an independent function of *VPS13A*, the well-studied role of PtdIns phosphates as regulators of actin cytoskeletal dynamics suggests that the reported effects of loss of *VPS13A* on the cytoskeleton may be a consequence of altered plasma membrane PtdIns(4)P levels [30]. In particular, a defining feature of ChAc is the appearance of acanthocytes. Many components of the spectrin/actin cytoskeleton that underlies the red blood cell membrane and controls cell shape are known to bind to PtdIns(4)P and PtdIns(4,5)P_2_[31,32]. Again, altered plasma membrane PtdIns(4)P pools in this cell type could account for the morphologic abnormalities seen in ChAc patients.

PC12 cells are induced to extend neurites in response to NGF treatment, which serves as an *in vitro* proxy for axonal and dendritic growth during neural development[27]. A *VPS13A* knockdown PC12 line displayed no significant differences in the rate of appearance or extension of neurites. However, after several days of growth, the neurites displayed morphological abnormalities, specifically blebs along the length of the neurites. The formation of these blebs, or beads, has been described previously and is considered a hallmark of neurite degeneration for both cultured primary neurons and PC12 cells[33,34]. Thus, although neurite outgrowth appears to be unaffected, the maintenance of the neurites is severely compromised in *VPS13* knockdown cells. Neurite degeneration could result from a variety of possible defects, a prominent one being defects in actin microfilament and/or microtubule cytoskeletal assembly[35,36]. The precise defect in neurite maintenance and whether there is any relationship between these effects in PC12 cells and the neural degeneration seen in ChAc patients remains to be determined.

In summary, we provide evidence that regulation of PtdIns(4)P levels in specific cellular membranes is a conserved function of the Vps13 family of proteins. The loss of this activity may account for the alteration in red blood cell morphology exhibited in ChAc. Whether the neuromuscular degeneration seen in ChAc patients is a long term consequence of altered PtdIns(4)P levels in neuronal cells or results from loss of some other aspect of *VPS13A* activity is an important question that remains to be explored.
